# Alpha-enolase promotes cell glycolysis, growth, migration, and invasion in non-small cell lung cancer through FAK-mediated PI3K/AKT pathway

**DOI:** 10.1186/s13045-015-0117-5

**Published:** 2015-03-08

**Authors:** Qiao-Fen Fu, Yan Liu, Yue Fan, Sheng-Ni Hua, Hong-Ying Qu, Su-Wei Dong, Rui-Lei Li, Meng-Yang Zhao, Yan Zhen, Xiao-Li Yu, Yi-Yu Chen, Rong-Cheng Luo, Rong Li, Li-Bo Li, Xiao-Jie Deng, Wei-Yi Fang, Zhen Liu, Xin Song

**Affiliations:** Cancer Center, Traditional Chinese Medicine-Integrated Hospital of Southern Medical University, Guangzhou, Guangdong People’s Republic China; Cancer Research Institute of Southern Medical University, Guangzhou, Guangdong People’s Republic China; Department of Pathology, Basic School of Guangzhou Medical University, Guangzhou, Guangdong People’s Republic China; Department of Cancer Biotherapy Center, Third Affiliated Hospital of Kunming Medical University (Tumor Hospital of Yunnan Province), Kunming, Yunnan People’s Republic China

**Keywords:** ENO1, NSCLC, Glycolysis, Cell proliferation, FAK/PI3K/AKT, EMT

## Abstract

**Background:**

During tumor formation and expansion, increasing glucose metabolism is necessary for unrestricted growth of tumor cells. Expression of key glycolytic enzyme alpha-enolase (ENO1) is controversial and its modulatory mechanisms are still unclear in non-small cell lung cancer (NSCLC).

**Methods:**

The expression of ENO1 was examined in NSCLC and non-cancerous lung tissues, NSCLC cell lines, and immortalized human bronchial epithelial cell (HBE) by quantitative real-time reverse transcription PCR (qRT-PCR), immunohistochemistry, and Western blot, respectively. The effects and modulatory mechanisms of ENO1 on cell glycolysis, growth, migration, invasion, and *in vivo* tumorigenesis and metastasis in nude mice were also analyzed.

**Results:**

ENO1 expression was increased in NSCLC tissues in comparison to non-cancerous lung tissues. Similarly, NSCLC cell lines A549 and SPCA-1 also express higher ENO1 than HBE cell line in both mRNA and protein levels. Overexpressed ENO1 significantly elevated NSCLC cell glycolysis, proliferation, clone formation, migration, and invasion *in vitro*, as well as tumorigenesis and metastasis *in vivo* by regulating the expression of glycolysis, cell cycle, and epithelial-mesenchymal transition (EMT)-associated genes. Conversely, ENO1 knockdown reversed these effects. More importantly, our further study revealed that stably upregulated ENO1 activated FAK/PI3K/AKT and its downstream signals to regulate the glycolysis, cell cycle, and EMT-associated genes.

**Conclusion:**

This study showed that ENO1 is responsible for NSCLC proliferation and metastasis; thus, ENO1 might serve as a potential molecular therapeutic target for NSCLC treatment.

**Electronic supplementary material:**

The online version of this article (doi:10.1186/s13045-015-0117-5) contains supplementary material, which is available to authorized users.

## Introduction

Lung cancer arises from the bronchial mucosal epithelium and it is the leading cause of cancer mortality worldwide. Non-small cell lung cancer (NSCLC) is the most commonly diagnosed type of lung cancer, accounting for approximately 85% of all cases. Although the continuous progress has been made for surgical resection, chemotherapy, and radiation therapy [1-3], prognoses have not significant improved. In recent years, molecular targeted therapy [4,5] has become the most prevalent approach. Therefore, the understanding of the molecular alterations in NSCLC and their pathways is significant for molecular targeted therapy.

During tumor formation and expansion, increasing glucose metabolism is necessary for the unrestricted growth of tumor cells [6]. Distributed in a variety of tissues, α-enolase (ENO1) was originally described as an enzyme responsible for the glycolytic pathway [7]. In addition to its glycolytic function, accumulating evidence has demonstrated that ENO1 is a multifunctional protein involved in several biological and pathophysiological processes depending on its cellular localization [8]. The molecular weight of ENO1 protein is 48 kDa. It is expressed in the cytoplasm and considered as an oncogene in tumor pathogenesis. However, another transcript of ENO1 can be translated into a 37-kDa c-Myc promoter-binding protein (MBP-1), which represses transcription and is localized in the nucleus [9-11].

Overexpression of ENO1 has been previously demonstrated in several types of tumors including NSCLC [12]. However, investigators have reported conflicting results. Some researchers have shown that the expression of ENO1 was upregulated in NSCLC tissues and was associated with poorer clinical outcomes [13,14]. On the contrary, Chang Y.S. *et al*. demonstrated that the levels of ENO1 protein were significantly decreased in NSCLC [15] and overexpression of ENO1 inhibited epithelial-mesenchymal transition (EMT) in the A549 cell line [16]. Therefore, neither expression nor the functional mechanisms of ENO1 in NSCLC have been clearly established.

In order to further validate the role of ENO1 and its molecular basis in NSCLC, we analyzed the expression of ENO1 in human NSCLC tissues and cell lines, as well as its effects on cell glycolysis, growth, migration, and invasion *in vitro* and tumorigenicity and metastasis *in vivo*. Our study showed that ENO1 is overexpressed in NSCLC tissues, and upregulated ENO1 promotes cell glycolysis, proliferation, migration, invasion, and tumorigenicity via the FAK/PI3K/AKT pathway. This is the first report of the molecular mechanisms of ENO1 in NSCLC, even more in-depth than our previous report of ENO1 in glioma [17].

## Results

### ENO1 is highly expressed in NSCLC

**Figure 1:**
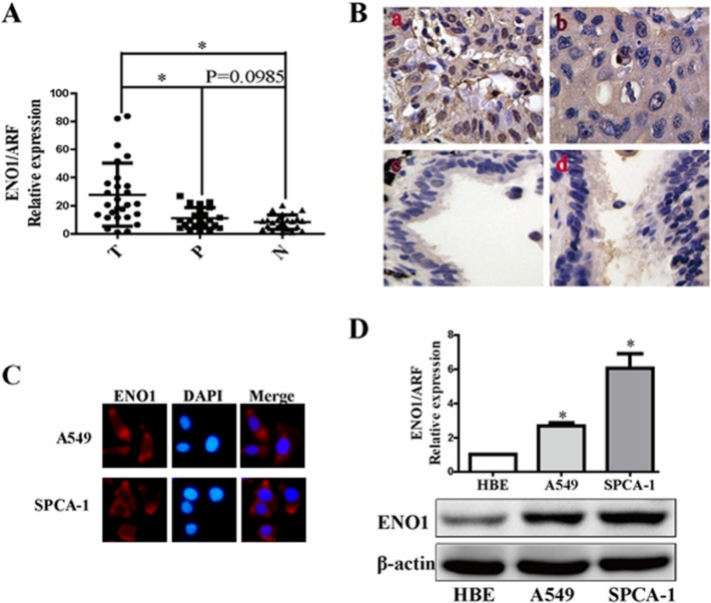
**ENO1 is highly expressed in NSCLC. (A)** The expression of ENO1 mRNA in NSCLC (T) and control tissues (P, N) was analyzed by quantitative RT-PCR. Data show the mean ± SD of three independent experiments (*P < 0.05). **(B)** The expression of ENO1 protein in NSCLC and non-cancerous lung specimens was analyzed by immunohistochemistry. **a**, **b)** Staining of ENO1 in primary NSCLC tissues. **c**, **d)** Staining of ENO1 in non-cancerous lung tissues. **(C)** Immunofluorescence staining of ENO1 in A549 and SPCA-1 cells. **(D)** Quantitative RT-PCR and Western blot showing the expression of ENO1 mRNA and protein in two NSCLC cell lines and immortalized normal bronchial epithelial cell line (HBE). Bars show the mean ± SD of three independent experiments (*P < 0.05). β-Actin served as protein loading control.

Quantitative real-time reverse transcription PCR (qRT-PCR) was used to measure the expression of ENO1 mRNA in 26 fresh primary NSCLC tissues (T), their corresponding para-cancer lung tissues (P), and their corresponding non-cancerous lung tissues (N). The ENO1 mRNA expression level was increased in NSCLC tissues in comparison to non-cancerous lung tissues (*P* < 0.05) (Figure 1A). The expression levels and subcellular localization of ENO1 protein in 55 paraffin-embedded primary NSCLC specimens and 17 paraffin-embedded non-cancerous lung specimens were measured by immunohistochemical staining (Figure 1B). Expression of ENO1 both in the cytoplasm and nucleus (MBP-1) were observed in NSCLC tissue, but as ENO1 is only known to localize in the cytoplasm, only this specific staining was evaluated. ENO1 protein was highly expressed in NSCLC tissues compared to non-cancerous lung samples (*P* = 0.019) (Table 1). Further, ENO1 was observed to express in the cytoplasm but not in the nucleus in NSCLC A549 and SPCA-1 cells by immunofluorescence assay (Figure 1C), and its upregulated expression levels in mRNA and protein were also found in both two cells compared to immortalized human bronchial epithelial cell line HBE (Figure 1D).

**Table 1:** **Upregulation of ENO1 protein in NSCLC specimens compared to non-cancerous lung specimens**

**Group**	**Cases (** ***n*** **)**	**Protein expression (** ***n*** **)**		***P*** **value**
		**Positive expression**	**Negative expression**	
NSCLC	55	34	21	0.019
Normal	17	5	12	

Chi-square test.

### Stable ENO1-overexpressed and ENO1-suppressed NSCLC cells as well as transient ENO1-suppressed NSCLC cells were constructed

**Figure 2:**
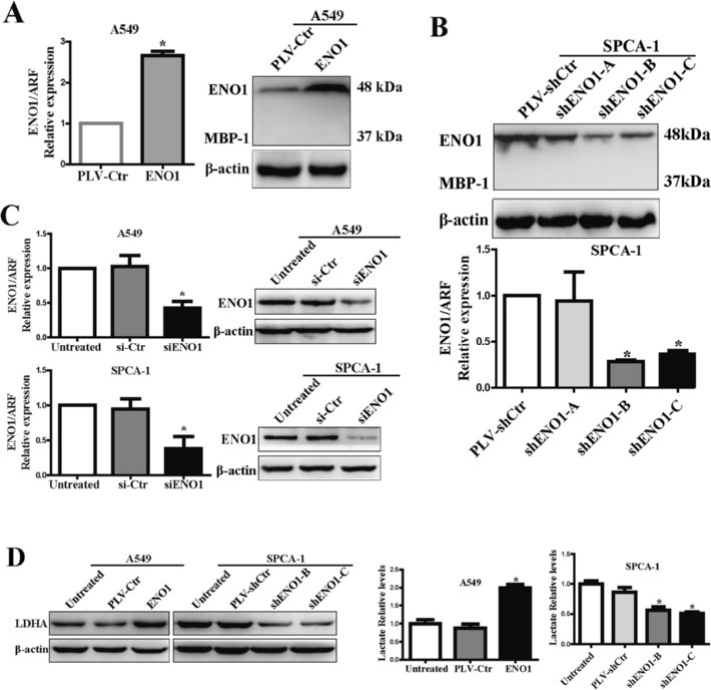
**The levels of ENO1 and glycolysis in ENO1-overexpressed and ENO1-suppressed NSCLC cells. (A)** Stably upregulated ENO1 by full-length ENO1 increased the expression of ENO1 in A549 cells by qRT-PCR and Western blotting. **(B)** Stably knocking down ENO1 by shRNA reduced the expression of ENO1 in SPCA-1 cells by qRT-PCR and Western blotting. **(C)** Transiently knocking down ENO1 by siRNA reduced the expression of ENO1 by qRT-PCR and Western blotting. **(D)** Western blotting showing the protein expression level of LDHA in ENO1-overexpressed A549 cells and ENO1-suppressed SPCA-1 cells. The histograms showed the relative levels of lactate in NSCLC with ENO1 overexpression and silencing. All of the experiments were repeated at least three times. β-Actin served as a loading control. Bars show the mean ± SD; (*P < 0.05).

Since ENO1 expression is higher in SPCA-1 than in A549 (Figure 1D), we firstly used lentivirus-mediated full-length ENO1-GFP (ENO1) to constitutively overexpress ENO1 in A549 cells in order to assess its role in NSCLC. The result showed that ENO1 expression was obviously upregulated in A549-ENO1 cells compared to its control PLV-Ctr cells, and the expression of MBP-1 was not observed (Figure 2A). Further, three lentiviral short hairpin RNA (shRNA) vectors were used to specifically and stably knock down the expression of ENO1 in the SPCA-1 cell line, and the expression levels of ENO1 and MBP-1 were determined by qRT-PCR and Western blot. The result indicated that ENO1 expression was obviously downregulated in shENO1-B and shENO1-C cells compared to their respective control PLV-scrambled control shRNA (shCtr) cells (Figure 2B). Similarly, the expression of MBP-1 was not observed in SPCA-1 cells (Figure 2B). To further evaluate the functional significance of ENO1 on NSCLC, small-interfering RNA (siRNA) was used to transiently silence ENO1 in A549 and SPCA-1 cells, and the expression of ENO1 were validated by qRT-PCR and Western blot (Figure 2C).

### ENO1 regulates the glycolysis in NSCLC cells

To assess the glycolysis changes triggered by ENO1, we used Western blot to detect the expression of lactate dehydrogenase A (LDHA) in ENO1-overexpressed A549 cells and ENO1-suppressed SPCA-1 cells. We found that the protein level of LDHA was markedly increased in ENO1-overexpressed A549 cells. In contrast, the expression of LDHA was obviously decreased in ENO1-suppressed SPCA-1 cells. To further confirm our results, we examined the level of lactate production in ENO1-overexpressed A549 cells and ENO1-suppressed SPCA-1 cells. Consistent with the results of the Western blot, ENO1-overexpressed A549 cells produced a more amount of lactate compared to its control cells and untreated cells. Conversely, the production of lactate was significantly less in ENO1-suppressed SPCA-1 cells than in its control cells and untreated cells, suggesting the involvement of ENO1 in inducing the glycolysis of NSCLC (Figure 2D).

### ENO1 promotes cell proliferation, clone formation *in vitro*, and tumorigenicity *in vivo*

**Figure 3:**
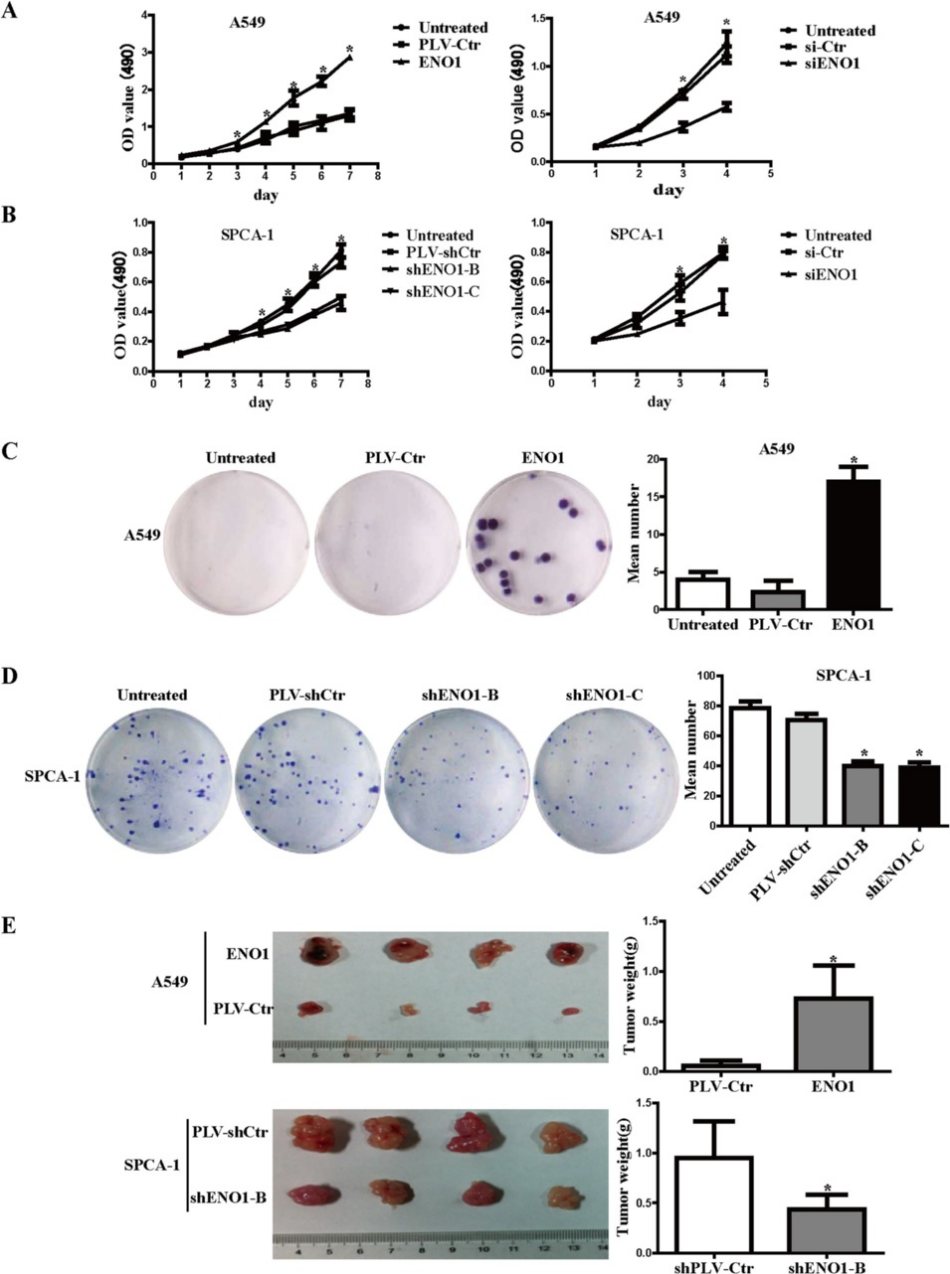
**ENO1 promotes cell proliferation** ***in vitro*** **and tumorigenicity** ***in vivo*** **. (A)** *In vitro* viability of A549 cell was increased in ENO1-overexpressed cells and was reduced in ENO1-suppressed cells compared to control cells by MTT assay. **(B)** *In vitro* viability of SPCA-1 cell was decreased in ENO1-suppressed cells compared to control cells by MTT assay. **(C)** *In vitro* proliferative ability of A549 cells was significantly increased in ENO1-overexpressed cells compared to control cells by clone formation assay. **(D)** *In vitro* proliferative ability of SPCA-1 cells was significantly decreased in ENO1-suppressed cells compared to control cells by clone formation assay. **(E)** *In vivo* tumorigenicity of A549 cells in nude mice was significantly increased in ENO1-overexpressed cells compared to PLV-Ctr cells. *In vivo* tumorigenicity of SPCA-1 cells in nude mice was significantly decreased in ENO1-suppressed cells compared to PLV-shCtr cells (*N* = 6 per group). One-way ANOVA was used for MTT assay and plate clone formation. Data are presented as mean ± SD for three independent experiments (*P < 0.05).

Next we assessed the effect of ENO1 expression on A549 cell growth *in vitro*. The growth curves determined by 3-(4, 5-dimethylthiazol-2-yl)-2,5-diphenyltetrazolium bromide (MTT) assays showed that overexpressed ENO1 significantly elevated cell viability compared to its control cells and untreated cells. MTT assays also showed that transiently suppressed ENO1 significantly decreased cell viability in A549 cells (Figure 3A). Colony formation assays showed that overexpressed ENO1 significantly increased cell proliferation compared to its control cells and untreated cells (Figure 3C). On the contrary, suppressed ENO1 expression in SPCA-1 cells significantly inhibited cell viability (Figure 3B) and clone formation (Figure 3D). To confirm the growth effect of ENO1 *in vivo*, we performed an *in vivo* tumorigenesis study by inoculating A549 with or without ENO1 overexpression and SPCA-1 cells with or without ENO1 knockdown into nude mice. Mice were sacrificed 15 days after inoculation, with average tumor weights of 0.059 ± 0.016 vs 0.73 ± 0.12 g in PLV-Ctr vs A549-ENO1 group and 0.95 ± 0.13 vs 0.435 ± 0.051 g in PLV-shCtr vs shENO1-B group, respectively (*P* < 0.01) (Figure 3E). These results suggest that ENO1 significantly promotes cell growth *in vitro* and *in vivo*.

### ENO1 promotes cell migration and invasion

**Figure 4:**
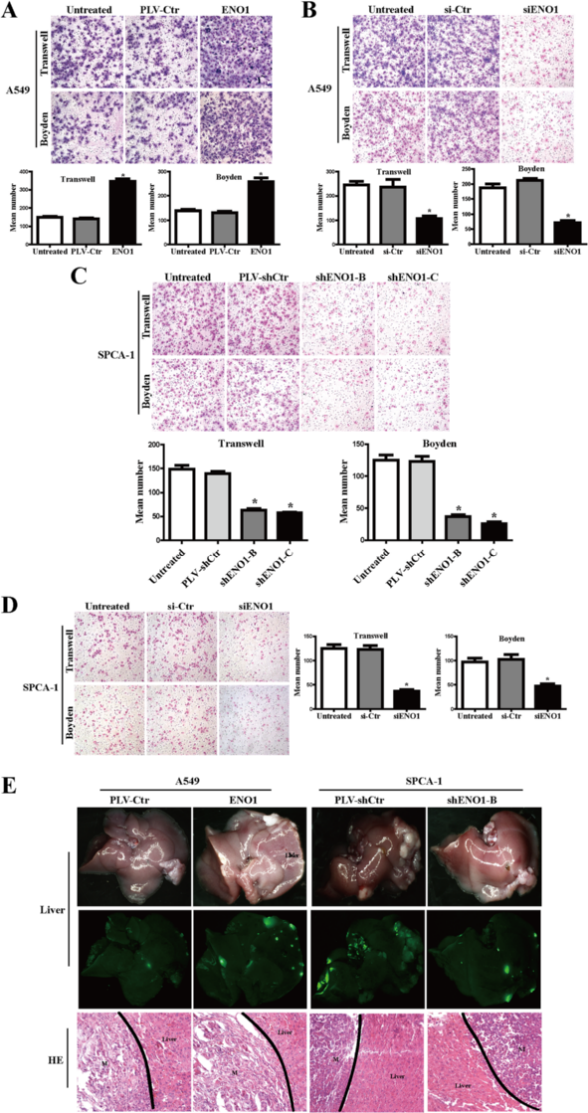
**ENO1 promotes cell migration and invasion. (A)** Stably upregulated ENO1 elevated the migration and invasion of A549 cells *in vitro*. **(B)** Transiently knocking down ENO1 reduced the migration and invasion of A549 cells *in vitro*. **(C, D)** Stable and transient downregulated ENO1 reduced the migration and invasion of SPCA-1 cells *in vitro*. **(E)** External optical fluorescence images of liver were obtained 40 days after spleen injection. Representative photographs of H&E staining of metastatic cancer tissues (M) are shown. Data are presented as mean ± SD for three independent experiments (*P < 0.05).

To examine the effect of ENO1 on cell migration and invasion, a transwell apparatus and Boyden chamber coated with Matrigel were used. After 10-h incubation, an elevated number of migrated cells were observed in A549-ENO1 compared to its control cells and untreated cells (*P* < 0.01) (Figure 4A). On the contrary, stably suppressed ENO1 expression in SPCA-1 cells inhibited cell migration and invasion in both shENO1-B and shENO1-C cell groups compared to their respective control cells and untreated cells (*P* < 0.01) (Figure 4C). Furthermore, similar results were also observed in siRNA-mediated suppression of ENO1 in NSCLC cells (Figure 4B, D). To further assess the effect of ENO1 on NSCLC metastasis *in vivo*, ENO1-overexpressed A549 cells, ENO1-suppressed SPCA-1 cells, and their control cells were independently injected into the spleens of nude mice. Fluorescence images showed that a large amount of intra-liver metastasis nodules was generated in the mice injected with A549-ENO1 cells, while a few small clusters were observed in A549 PLV-Ctr cells. In addition, a few small nodules were observed in SPCA-1 shENO1-B cells, while a variety of large clusters were observed in SPCA-1 PLV-shCtr cells. These are consistent with the hematoxylin and eosin (H&E)-stained liver sections (Figure 4E). Similar to the results *in vitro*, ENO1 promotes the metastasis of NSCLC cells.

### ENO1 regulates the expression of cell cycle and EMT-associated genes in NSCLC

**Figure 5:**
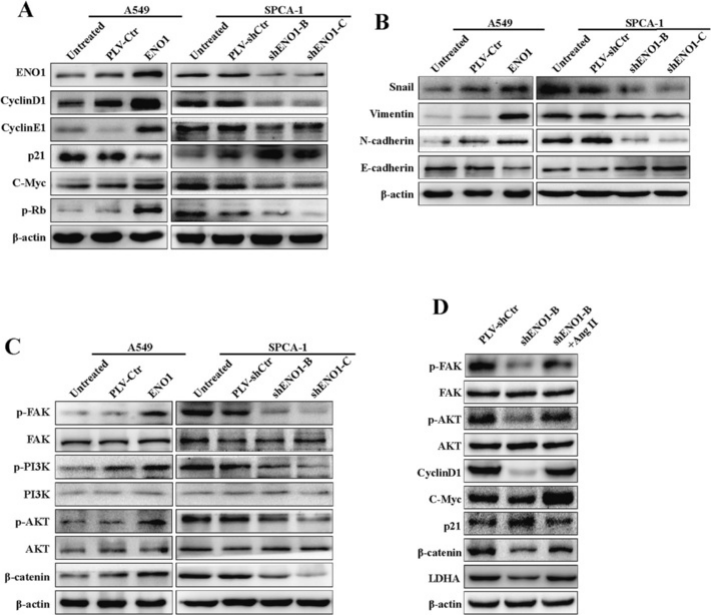
**ENO1 regulates the expression of cell cycle and EMT-associated genes via FAK/PI3K/AKT pathway in NSCLC cells. (A)** In A549 cells, overexpressed ENO1 increased the levels of p-Rb (ser 780) and oncogenic cell cycle regulators cyclin D1, cyclin E1, and c-Myc and decreased the expression of tumor suppressor p21. Conversely, downregulated ENO1 expression in SPCA-1 cells inhibited the expression of these proteins in addition to p21. **(B)** In A549 cells, overexpressing ENO1 increased the expression of EMT-marker genes including snail, vimentin, and N-cadherin and decreased the expression of E-cadherin. In SPCA-1 cells, suppressing ENO1 expression decreased the expression of these proteins in addition to E-cadherin. **(C)** In A549 cells, upregulated ENO1 increased levels of β-catenin, phos-FAK, PI3K, and AKT, but not their total protein levels; in SPCA-1 cells, reduced ENO1 expression reduced the levels of β-catenin, phos-FAK, PI3K, and AKT, but not their total protein levels. **(D)** ENO1-suppressed SPCA-1 cells were treated with Ang II (1 μmol/l) for 12 h to activate the phosphorylation of FAK, then cellular p-FAK, p-AKT, LDHA, cyclin D1, c-Myc, p21, and β-catenin were assessed by Western blot. β-Actin served as a loading control. All of the experiments were repeated at least three times.

**Figure 6:**
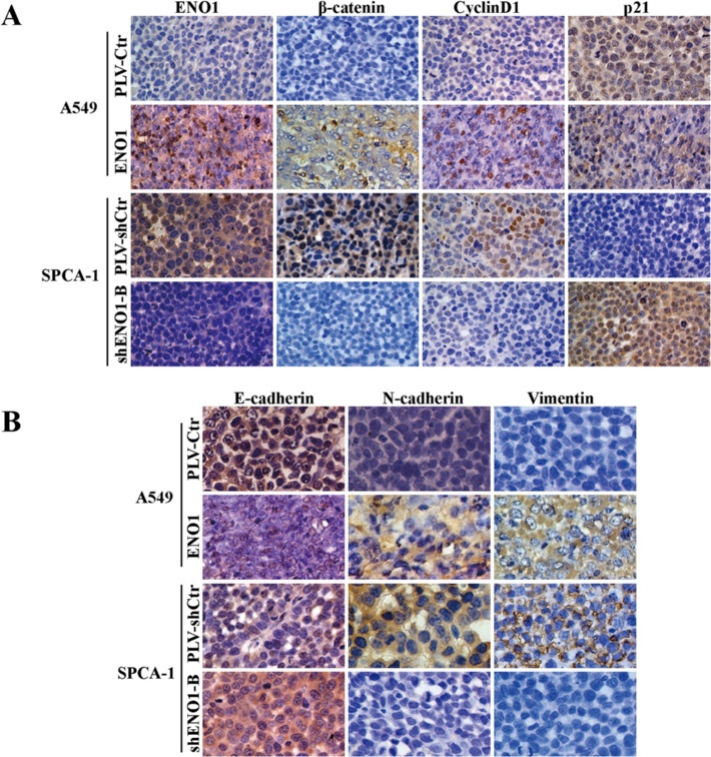
**ENO1 regulates the expression of cell cycle and EMT-associated genes of NSCLC cells.** Immunohistochemical (IHC) staining of ENO1, β-catenin, cyclin D1, p21 **(A)**, and EMT-associated genes **(B)** including E-cadherin, N-cadherin, and vimentin in subcutaneous tumors of mice injected with PLV-Ctr vs A549-ENO1, PLV-shCtr vs shENO1-B SPCA-1 cells.

To further study the mechanism by which ENO1 regulates cell proliferation, migration, and invasion, the protein levels of cell cycle and EMT-associated genes were examined in A549 and SPCA-1 cells with stably overexpressed or suppressed ENO1. In ENO1 stably overexpressing A549 cells, activation of p-Rb (ser 780) was increased as well as the elevated expression of cyclin D1, cyclin E1, and c-Myc. In contrast, the expression of p21 was inhibited. Stably knocking down endogenous ENO1 expression in SPCA-1 inhibited the activation of p-Rb (ser 780), and the expression of cyclin D1, cyclin E1, and c-Myc were decreased, while levels of p21 were upregulated (Figure 5A). We also found that upregulated ENO1 expression elevated the expression of EMT marker genes including snail, vimentin, and N-cadherin, yet inhibited E-cadherin in A549 cells. Conversely, downregulated ENO1 expression in SPCA-1 cells inhibited the expression of these proteins and elevated E-cadherin expression (Figure 5B). Similar changes in cell cycle regulators cyclin D1 and p21 as well as EMT-associated genes including E-cadherin, N-cadherin, and vimentin were observed in tumor tissues by IHC (Figure 6A, B). However, stable downregulated or upregulated ENO1 did not induce any epithelial to mesenchymal morphology transition changes in A549 or SPCA-1 cells (Additional file 1: Figure S1).

### ENO1 regulates FAK-mediated PI3K/AKT pathway to promote cell glycolysis, proliferation, migration, and invasion

**Figure 7:**
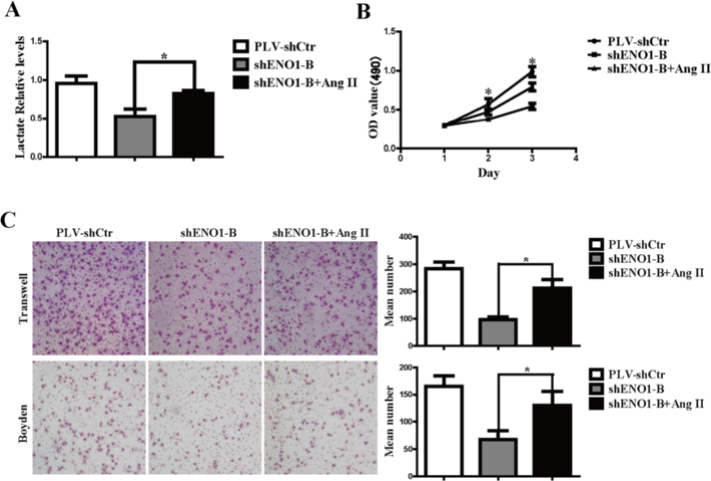
**Ang II treatment reverses the inhibitory effect of stably downregulated ENO1 in SPCA-1 cells.** ENO1-suppressed SPCA-1 cells were treated with Ang II (1 μmol/l) for 12 h to activate the phosphorylation of FAK, and cell viability, migration, and invasion were examined by MTT assay, transwell apparatus, and Boyden chamber, respectively. **(A)** The inhibiting effect of stably downregulated ENO1 in glycolysis was reversed by Ang II treatment. **(B)** The inhibiting effect of stably downregulated ENO1 in cell proliferation was reversed by Ang II treatment. **(C)** The inhibiting effect of stably downregulated ENO1 in cell migration and invasion was reversed by Ang II treatment. Data are presented as mean ± SD for three independent experiments (*P < 0.05).

PI3K/AKT has been reported to be a key signal pathway promoting cell proliferation and EMT and can be modulated by FAK [18]. We found that overexpression of ENO1 significantly increased levels of β-catenin and phosphorylated FAK, PI3K, and AKT, but not their total protein levels (Figure 5C). Suppression of ENO1 had the opposite effect on the FAK/PI3K/AKT pathway. To further study the mechanism by which ENO1 regulates cell glycolysis, proliferation, migration, and invasion, ENO1-suppressed SPCA-1 cells were treated with human angiotensin II (Ang II) to induce the phosphorylation of FAK [19]. Ang II treatment reversed the effects of ENO1 knockdown on cell glycolysis, viability, migration, and invasion (Figure 7A–C). We observed a consistent effect on the FAK/PI3K/AKT pathway after Ang II treatment of ENO1-suppressed SPCA-1 cells whereby levels of p-AKT, LDHA, cyclin D1, c-Myc, p21, and β-catenin were restored (Figure 5D). These results implied that ENO1 is an upstream signal factor modulating the FAK/PI3K/AKT pathway in NSCLC, and ENO1 regulates FAK/PI3K/AKT pathway to promote cell glycolysis, proliferation, migration, and invasion.

## Discussion

Upregulated expression of ENO1 has been detected in several cancers, such as glioblastoma [20], head and neck cancer [21], pancreatic cancer [22], and prostate cancer [23]. However, the role of ENO1 in NSCLC is still controversial [13-16], which needs to be further identified. In this study, we confirmed that the expression of ENO1 mRNA and protein was frequently overexpressed in NSCLC tissues compared to non-cancerous lung tissues as well as in NSCLC cells compared to HBE cells. These results are consistent with Chang *et al*.’s report supporting an oncogenic role for ENO1 in NSCLC [13], but not Chang’s study [15].

In order to evaluate the function of ENO1 and eliminate the influence of MBP-1 on NSCLC, we firstly performed an immunofluorescence and observed that ENO1 was expressed in the cytoplasm but not in the nucleus (MBP-1) in A549 and SPCA-1 cells. Furthermore, we also found that MBP-1 was not expressed by Western blot assay in A549 and SPCA-1 cells. The abovementioned results suggested that both two cells could be used as well-defined models to evaluate the function of ENO1 on NSCLC. Further, stable ENO1-overexpressed A549 cells and stable ENO1-suppressed SPCA-1 cells as well as transient ENO1-suppressed A549 and SPCA-1 cells were respectively constructed, which was used to investigate the role of ENO1 in NSCLC.

ENO1 was originally described as an enzyme responsible for the glycolytic pathway. To further assess the effect of ENO1 on NSCLC cells, we analyzed the glycolysis changes triggered by ENO1 and found that overexpressed and suppressed ENO1 respectively increased and decreased the production of lactate. These data suggested that ENO1 was involved in inducing glycolysis in NSCLC.

Previous studies have demonstrated that ENO1 overexpression was positively associated with progression and poor prognosis in neuroendocrine tumors, neuroblastoma, pancreatic cancer, prostate cancer, cholangiocarcinoma, thyroid carcinoma, hepatocellular carcinoma, and breast cancer [14,21-27]. Further, ENO1 has been shown to promote cell proliferation, cycle progression, migration, and invasion [14,21-33], which suggests that ENO1 functions as an oncogene in tumor pathogenesis. In this study, we found that overexpressed ENO1 significantly elevated cell proliferation and clone formation *in vitro* as well as tumorigenesis *in vivo*. Furthermore, we also observed that overexpressed ENO1 induced cell migration, invasion, and metastasis in NSCLC. Our results are consistent with previous reports in other tumors that ENO1 functions as an oncogene [13,14,29,34] but are in contrast with Zhou *et al*.’s report that ENO1 overexpression suppressed EMT in NSCLC A549 cell line [16].

The biological functions of ENO1 found in this study provide a mechanistic basis for the pathological and clinical observations. When we examined the key regulators of the glycolysis and cell cycle at the G1-S phase transition, we discovered that suppression of ENO1 inhibited the expression of LDHA, c-Myc, cyclin D1, p-Rb, and cyclin E1 while elevating the expression of p21, which promoted cell glycolysis and proliferation of NSCLC. EMT is regarded as a key event in tumor migration and invasion progression. In this study, we further examined the expression of EMT marker genes and found that knocking down ENO1 expression induced the protein levels of E-cadherin while suppressing the expression of snail, vimentin, and N-cadherin in NSCLC cells. These results are consistent with our previous report of ENO1 in glioma [17]. However, ENO1 overexpression did not lead to any changes from epithelial to mesenchymal transition in NSCLC cells.

PI3K/AKT is a key signal mediator during carcinogenesis [35,36], and its activation induces glycolysis [37-39] and c-Myc-mediated cell cycle transition [40] and promotes the progression of EMT [37,41]. In addition, c-Myc has also been shown to regulate energy metabolism by regulating LDHA in tumor [42]. We hypothesized that oncogenic ENO1 functions through the PI3K/AKT pathway in NSCLC. We found that suppressed ENO1 significantly decreased the protein levels of β-catenin and phosphorylated PI3K and AKT, but not their total protein levels in SPCA-1 cells, which is similar to our previous report in glioma [17]. Interestingly, we examined the protein levels of FAK, an upstream signal factor of the PI3K/AKT pathway, and found that suppressed ENO1 significantly decreased levels of phosphorylated FAK, but not its total protein levels. We speculated that ENO1 regulates cell glycolysis, proliferation, migration, and invasion through FAK-mediated PI3K/AKT pathway in NSCLC. To further clarify the specific mechanism, Ang II, an activating agent of phosphorylated FAK [19], was used to treat ENO1-suppressed SPCA-1 cells. We observed that not only the production of lactate, cell viability, migration, and invasion was restored but also the expression levels of p-FAK, p-AKT, LDHA, cyclin D1, c-Myc, p21, and β-catenin were rescued. These results demonstrated that suppressed ENO1 inhibited cell glycolysis, proliferation, migration, and invasion by inactivating FAK-mediated PI3K/AKT pathway in NSCLC. Thus, ENO1 may be a potential therapeutic target for NSCLC treatment. Furthermore, nanotechnology has provided a good platform for cancer targeted therapy based on nanoparticle unique properties. Therefore, we wish to develop a nanoparticle formulation modified with tumor-targeting single-chain antibody fragment (scFv) for systemic delivery of siRNA-ENO1 in the future [43], which may make it possible that ENO1 serves as a molecular therapeutic target for NSCLC treatment.

## Conclusions

In summary, ENO1 is overexpressed in NSCLC, promoting cell glycolysis, proliferation, migration, invasion, and tumorigenesis by activating the FAK-mediated PI3K/AKT pathway and further modulating their downstream signal molecules. To our knowledge, this is the first report of the molecular mechanisms of ENO1 in NSCLC and even more in-depth than our previous report of ENO1 in glioma [17]. Our study demonstrates that ENO1 may be a potential therapeutic target for NSCLC treatment.

## Materials and methods

### Cell culture and sample collection

A549 line was obtained from ATCC Bioresource Center, and SPCA-1 and HBE were purchased from Chinese Academy of Sciences Cell Bank (Shanghai, China). A549 cells were cultured in Dulbecco’s modified Eagle’s medium (DMEM, HyClone, Logan, UT) supplemented with 10% fetal bovine serum (FBS) (ExCell, Shanghai, China); SPCA-1 was cultured in RPMI 1640 medium (HyClone, Logan, UT) supplemented with 10% FBS (ExCell, Shanghai, China). HBE, an immortalized human bronchial epithelial cell line, was grown in DMEM (HyClone, Logan, UT) supplemented with 20% FBS (ExCell, Shanghai, China). All cell lines were maintained in a humidified chamber with 5% CO_2_ at 37°C. Twenty-six (26) surgical resected fresh primary NSCLC tissues and paired para-cancer lung tissues as well as non-cancerous lung tissues (5 cm away from tumor edge), 55 paraffin-embedded primary NSCLC specimens, and 17 paraffin-embedded non-cancerous lung specimens were obtained from the Third Affiliated Hospital of Kunming Medical University (Yunnan, China). Patients with a diagnosis of relapse and who had received preoperative radiation, chemotherapy, or biotherapy were excluded from the study to avoid any changes in tumor marker determination due to the effect of the treatment. The clinical processes were approved by the Ethics Committees of the Third Affiliated Hospital of Kunming Medical University, and patients provided informed consent. Demographic and clinical data were obtained from the patients’ medical records.

### RNA isolation, RT-PCR, qRT-PCR, and primers

Total RNA was extracted from the cell lines and lung tissues using Trizol (Takara, Shiga, Japan). RNA (1 μg) was reverse transcribed into cDNA, and cDNA was used as a template to amplify with specific primers for sense: 5′-TCAATGGCGGTTCTCATGCT-3′ and for antisense: 5′-GCAGCTCCAGGCCTTCTTTA-3′. ARF5 was used as an internal control with primers for sense: 5′-ATCTGTTTCACAGTCTGGGACG-3′ and for antisense: 5′-CCTGCTTGTTGGCAAATACC-3′. Experiments were performed according to the manufacturer’s instructions (Takara, Shiga, Japan). PCR conditions were 95°C for 10 min to activate DNA polymerase, followed by 45 cycles of 95°C for 15 s, 60°C for 15 s, and 72°C for 15 s. Specificity of amplification products was determined by melting curve analysis. The qRT-PCR reactions for each sample were repeated three times. Independent experiments were done in triplicate.

### Immunohistochemistry and evaluation of staining

Immunohistochemistry was performed in primary NSCLC tissues, non-cancerous lung tissues, and mouse tumors according to a previous description [44] with rabbit polyclonal anti-ENO1 antibody (1:150; Proteintech, USA), anti-cyclin D1, p21 antibody (1:100; Epitomics, Burlingame, USA), β-catenin, N-cadherin, or E-cadherin antibody (1:100; Cell Signaling Technology, Danvers, USA).

Stained tissue sections were reviewed and scored independently by two investigators blinded to the clinical data. For cytoplasmic staining, the score was based on the sum of cytoplasm staining intensity and the percentage of stained cells. The staining intensity was scored as previously described (0–3) [44,45], and the percentage of positive staining areas of cells was defined as a scale of 0–3 (0: <10%, 1: 10%–25%, 2: 26%–75%, and 3: >76%). For nuclear staining, the staining score was defined based on the sum of nuclear staining intensity and the percentage of positive nuclear staining. Positive nuclear staining scores were defined as follows: 0: <20%, 1: 20%–49%, 2: 50%–79%, and 3: >80%. The sum of the staining intensity and staining extent scores (0–6) was used as the final staining score. For statistical analysis, a final staining score of 0 ~ 2 and 3 ~ 6 in cytoplasm or 0 ~ 3 and 4 ~ 6 in nucleus were respectively considered to be negative and positive expression levels. Expression of ENO1 in the nucleus was observed, but since ENO1 localizes in cytoplasm, only the cytoplasmic staining was evaluated.

### Immunofluorescence

Immunofluorescence was performed according to a previous study [46]. NSCLC cells were seeded on coverslips in six-well plate and cultured overnight. Subsequently, cells were fixed in 3.5% paraformaldehyde and permeabilized in KB solution and 0.2% Triton X-100 at room temperature. After the blocking solution was washed out, cells were incubated with a primary antibody (ENO1) (diluted in KB) for 30–45 min at 37°C and subsequently washed with KB twice. After incubating for 30–45 min at 37°C with secondary antibody (diluted in KB) and washing with KB again, the coverslips were then mounted onto slides with mounting solution containing 0.2 mg/ml DAPI and sealed with nail polish. Slides were stored in a dark box and observed under a fluorescent microscope.

### Western blot analysis, reagent, and antibodies

Western blotting was performed as described [47] with rabbit polyclonal anti-ENO1, LDHA antibody (1:1,000; Proteintech, USA), anti-cyclin D1, p21, cyclin E1, C-Myc antibody (1:1,000; Epitomics, Burlingame, USA), anti-CDK4 antibody (1:400; Santa Cruz Biotechnology, Santa Cruz, USA), anti-pRb (Ser780), FAK, p-FAK (Tyr397), AKT, p-AKT (Ser473), PI3K, p-PI3K (Tyr458), snail, β-catenin, N-cadherin, vimentin, and E-cadherin antibody (1:1,000; Cell Signaling Technology, Danvers, USA). An HRP-conjugated anti-rabbit IgG antibody was used as the secondary antibody (Zhongshan, Beijing, China). Signals were detected using enhanced chemiluminescence reagents (Pierce, Rockford, IL). Ang II was purchased from the Santa Cruz Biotechnology (Santa Cruz, USA).

### Transfection and infection

The full-length ENO1-GFP (ENO1), GFP empty vector (PLV-Ctr) lentiviruses were designed by Shanghai Genechem (Genechem, Shanghai, China). The preparation of lentiviruses expressing human ENO1 short hairpin RNA (shENO1-A, B, C) (Table 2) was performed using the pLVTHM-GFP lentiviral RNAi expression system [40]. NSCLC cell line A549 was infected with full-length ENO1-GFP or GFP empty vector lentiviruses. SPCA-1 cells were infected with shENO1-A, B, C or PLV-shCtr lentiviruses, and polyclonal cells with GFP signals were selected for further experiments using FACS flow cytometry. Total RNA was isolated, and levels of ENO1 mRNA were measured using real-time PCR analysis.

**Table 2:** **shRNA sequences for ENO1**

**shENO1**		**Sequence**
A	Sense	5′-CCGGAATGTCATCAAGGAGAAATATCTCGAGATATTTCTCCTTGATGACATTTTTTTG-3′
	Antisense	5′-AATTCAAAAAAATGTCATCAAGGAGAAATATCTCGAGATATTTCTCCTTGATGACATT-3′
B	Sense	5′-CCGGCGTGAACGAGAAGTCCTGCAACTCGAGTTGCAGGACTTCTCGTTCACGTTTTG-3′
	Antisense	5′-AATTCAAAACGTGAACGAGAAGTCCTGCAACTCGAGTTGCAGGACTTCTCGTTCACG-3′
C	Sense	5′-CCGGCCACTGTTGAGGTTGATCTCTCTCGAGAGAGATCAACCTCAACAGTGGTTTTTG-3′
	Antisense	5′-AATTCAAAAACCACTGTTGAGGTTGATCTCTCTCGAGAGAGATCAACCTCAACAGTGG-3′

### Transient transfection with siRNAs

siRNA for ENO1 was designed and synthesized by Guangzhou RiboBio (RiboBio Inc, China). The sequence of siENO1 is sense: 5′-GCAUUGGAGCAGAGGUUUAdTdT-3′ and anti-sense: 3′-dTdTCGUAACCUCGUCUCCAAAU-5′. The sequence of si-negative control (si-Ctr) was also designed by RiboBio (RiboBio Inc, China). Twenty-four hours prior to transfection, NSCLC cells A549 and SPCA-1 were plated onto a 6-well plate or a 96-well plate (Nest Biotech, China) at 30%–50% confluence. They were then transfected into cells using TurboFect TM siRNA Transfection Reagent (Fermentas, Vilnius, Lithuania) according to the manufacturer’s protocol. Cells were collected after 48–72 h for further experiments.

### Metabolic profiling

Metabolic profiles were obtained to assess the relative distribution of various cellular metabolites of NSCLC cells. Cells were collected and quickly frozen. Further sample preparation, metabolic profiling, peak identification, and curation were performed by Metabolon (Durham, NC, USA) using their described methods [48].

### MTT assay

The viability of cell proliferation was assessed using MTT assay according to our previous study [46]. Cells were seeded in 96-well plates at a density of 1,000 cells/well. Every 24 h for 7 days, 20 μl of MTT (5 mg/ml) (Sigma-Aldrich, St. Louis, MO) was added to each well and incubated for 4 h. Supernatants were removed, and 150 μl of dimethyl sulfoxide (DMSO) (Sigma-Aldrich, St. Louis, MO) was added to each well. The absorbance value (OD) of each well was measured at 490 nm. For each experimental condition, five parallel wells were assigned to each group. Experiments were performed thrice.

### Clone formation assay

Clone formation assay was performed according to our previous study [46]. Cells were seeded in 6-well culture plates at 100 cells/well. Each cell group had three parallel wells. After incubation for 14 days at 37°C, cells were washed twice with Hank’s solution and stained with hematoxylin solution. The number of colonies containing ≥50 cells was counted under a microscope. The clone formation efficiency was calculated as (number of colonies/number of cells inoculated) × 100%.

### Cell migration and invasion assays

*In vitro* cell migration and invasion assays were examined according to our previous study [46]. For cell migration assays, 1 × 10^5^ cells in a 100-μl medium without serum were seeded on a fibronectin-coated polycarbonate membrane insert in a transwell apparatus (Corning, USA). In the lower surface, 500 μl DMEM with 10% FBS was added as chemoattractant. After the cells were incubated for 10 h at 37°C in a 5% CO_2_ atmosphere, Giemsa-stained cells adhering to the lower surface were counted under a microscope in five predetermined fields (100×). All assays were independently repeated at least thrice. For cell invasion assays, the procedure was similar to the cell migration assay, except that the transwell membranes were pre-coated with 24 μg/ml Matrigel (R&D Systems, USA).

### *In vivo* tumorigenesis in nude mice

According to our previous study [17], a total of 1 × 10^6^ logarithmically growing A549 cells transfected with full-length ENO1 and PLV-Ctr vector, SPCA-1 cells transfected with shENO1-B, and the control PLV-shCtr vector (*N* = 6 per group) in 0.1 ml Hank’s solution were subcutaneously inoculated into the left-right symmetric flank of 4–6-week-old male BALB/c-nu/nu mice. The mice were maintained in a barrier facility on HEPA-filtered racks and fed an autoclaved laboratory rodent diet. All animal studies were conducted in accordance with the principles and procedures outlined in the National Institutes of Health Guide for the Care and Use of Animals under assurance number A3873-1. After 15 days, the mice were sacrificed, and their tumors were excised, weighed, and processed for histology.

### *In vivo* metastasis assays

*In vivo* metastasis assays were performed according to a previous study [46]. A total of 5 × 10^6^ cells were injected into nude mice (*n* = 5 for each group) through the spleen, respectively. The optical fluorescence images were visualized to monitor primary tumor growth and formation of metastatic lesions. Forty days later, all mice were killed, individual organs were removed, and metastatic tissues were analyzed by H&E staining.

### Statistical analysis

All data were independently repeated at least thrice. SPSS 13.0 and Graph Pad Prism 5.0 software were used for statistical analysis. One-way ANOVA or two-tailed Student’s *t*-test were applied to determine the differences between group *in vitro* analyses. The chi-squared test was used to determine the differences of ENO1 protein expression between NSCLC tissues and non-cancerous lung tissues of the lung. A *p* value of less than 0.05 was considered statistically significant.

## Additional file

Additional file 1: Figure S1. Stably upregulated ENO1 (A) or downregulated ENO1 (B) did not induce obvious epithelial to mesenchymal morphology transition changes in SPCA-1 or A549 cells.

## Abbreviations

ENO1: α-Enolase
MBP-1: c-Myc promoter-binding protein
NSCLC: Non-small cell lung cancer
EMT: Epithelial-mesenchymal transition
qRT-PCR: Quantitative real-time reverse transcription PCR
MTT: 3-(4 5-dimethylthiazol-2-yl)-2, 5-diphenyltetrazolium bromide
LDHA: Lactate dehydrogenase A
FAK: Protein tyrosine kinase 2
PI3K: Phosphoinositide 3-kinase
AKT: V-akt murine thymoma viral oncogene homolog 1
Ang II: Human angiotensin II
