# Supplementary figures and images for: Alpha-enolase promotes cell glycolysis, growth, migration, and invasion in non-small cell lung cancer through FAK-mediated PI3K/AKT pathway
Source: J Hematol Oncol. 2015 Mar 8;8:22. doi: 10.1186/s13045-015-0117-5 (PMC4359783; doi:10.1186/s13045-015-0117-5)

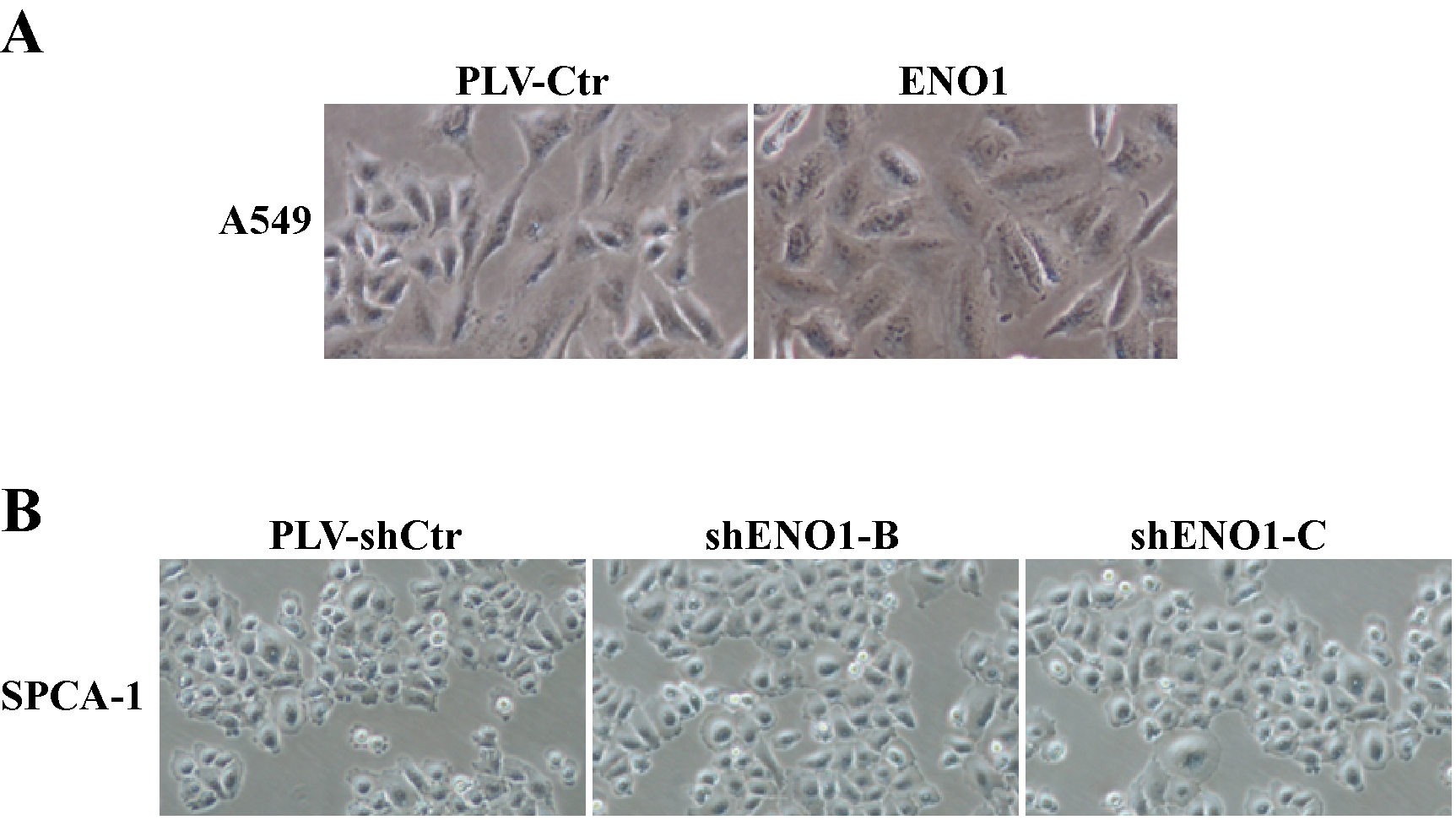

Supplement: Additional file 1: Figure S1. — Stably upregulated ENO1 (A) or downregulated ENO1 (B) did not induce obvious epithelial to mesenchymal morphology transition changes in SPCA-1 or A549 cells. [file 13045_2015_117_MOESM1_ESM.tiff]
